# The effectiveness of lifestyle interventions on ecological literacy: A contribution to the underlying mechanism in linguistic ecology

**DOI:** 10.1371/journal.pone.0287286

**Published:** 2023-06-29

**Authors:** Changchen Ha, Yang Chen, Jiaen Zhang, Shumin Dong

**Affiliations:** 1 School of International Studies, Sun Yat-sen University, Zhuhai, Guangdong, China; 2 School of Foreign Studies, South China Agricultural University, Guangzhou, Guangdong, China; 3 Center for Ecolinguistics, South China Agricultural University, Guangzhou, Guangdong, China; 4 College of Natural Resources and Environment, South China Agricultural University, Guangzhou, Guangdong, China; 5 School of Chinese Ethnic Minority Languages and Literature, Minzu University of China, Beijing, China; Northeast Normal University, CHINA

## Abstract

In today’s society, citizens’ ecological literacy (ecoliteracy) is critical for their understanding of sustainable development. This study used a questionnaire designed to quantitatively assess ecoliteracy from a linguistic ecology perspective. First, an underlying mechanism model for ecoliteracy was designed based on the results of previous studies. Then, the ecoliteracy level assessment scores of Guiyang inhabitants were combined with the respondents’ corresponding lifestyle characteristics to explore the effectiveness of interventions in affecting the participants’ ecoliteracy levels. The results showed that the formation and development of ecoliteracy is a dynamic and circular process that revolves around variables of independent, dependent, mediating, moderating and control. The various factors in the model interact and operate evenly along a particular path. As for the level of lifestyle characteristics, participants’ ecoliteracy levels had a statistically significant relationship with their attitudes regarding the importance of nature, participating in outdoor activities, and improving their ecoliteracy levels; as well as the frequencies regarding daily outdoor activity, the main activities in ecological areas, participation in volunteer activities, and use of ecological knowledge. The respondents with the highest levels of ecoliteracy had the most positive attitudes and engaged in ecological actions with the highest frequency. The lifestyle intervention features here are of great significance to the harmonious coexistence between humans and the natural environment and are also helpful for improving human health.

## Introduction

The United Nations has formulated the Sustainable Development Goals (SDGs), for guiding global development efforts from 2015 to 2030, to address the social, economic, and environmental aspects of development. Subsequently, researchers from different countries or regions began to solve specific problems in different ways, such as the significance of sustainable use of material resources for green growth [1], study of environmental quality and financial stress index in developing countries [2, 3], relationship between natural resources and economy in different regions [4–7], exploration of sustainable development in the United States and India [8–10]. Studies on ecological civilization and ecological cities are also reported in China [11, 12]. Ecological literacy (ecoliteracy) plays a very important role for sustainable development, which is the focus of our article.

“Literacy” is originally a concept from linguistic research, which focuses on “the ability to read and write” [13, 14]. With the development of interdisciplinary trends, the applied scope of literacy has continued to expand. It can be defined as “the knowledge or capability in a particular field or fields” [14]. The phrase “in a particular field or fields” means that it can be used in combination with a specific discipline. In a broader sense, ecoliteracy combines ecology and linguistics and has the same disciplinary foundation as the study of linguistic ecology [15]. However, ecoliteracy is not limited to these two disciplines but is, in fact, part of a larger set of terms [14, 16]. Researchers and scholars in different disciplinary fields, who apply different theories from different perspectives, produce significantly different concepts and frameworks (e.g., [14, 16–21]). Some studies focus on individuals’ levels of ecological knowledge [20, 22], while others consider attitudes toward ecological issues and content related to ecological behavior or other aspects of ecological topics [11, 16, 21, 23–25].

This study focused on an interdisciplinary perspective that combined ecology and linguistics (called linguistic ecology) to define ecoliteracy [11, 15]. Ecoliteracy is concerned with the ecologically sustainable development relationship between individual humans, humans and society, and humans and nature. It emphasizes the knowledge and ability of human beings in the ecological field [12]. Our ecoliteracy research framework included five dimensions [11]: ecological knowledge literacy (EKNL), ecological awareness literacy (EAWL), ecological ethics literacy (EETL), ecological emotional literacy (EEML), and ecological behavioral literacy (EBEL).

In the future, ecoliteracy will play a vital role in human survival and development. People will be required to have the ability to learn and understand the concepts and basic principles of ecology and live a sustainable life, accordingly implying that ecoliteracy is no longer unique to ecologists. In the work of political scientists, business leaders, professionals, or in education at all levels, ecoliteracy is an important component and key skill [26]. Previous studies on ecoliteracy have focused primarily on the theoretical research aspects of developing its connotation, ecosystems, sustainability, and interdisciplinary aspects [14,15, 17, 27–29]. However, relatively little attention has been paid to assessing individuals’ levels of ecoliteracy and cultivation of ecoliteracy [11, 20, 21, 23, 30, 31]. Research on the combination of ecoliteracy and lifestyle characteristics is even rarer [32, 33].

Therefore, this study focused on lifestyle interventions for ecoliteracy. First, the underlying mechanisms of ecoliteracy were explored from the theoretical perspective of linguistic ecology. Lifestyle characteristics as the core concept were then examined and the inhabitants of Guiyang City, one of China’s top ten ecologically advanced cities, were used as subjects for a case study. The main purpose was to discover differences in the levels of ecoliteracy among Guiyang inhabitants with different lifestyle characteristics. This article addressed two specific research questions: (1) What is the underlying mechanism of ecoliteracy in linguistic ecology? (2) Are there any differences in the levels of ecoliteracy among Guiyang’s inhabitants with different lifestyle characteristics?

## Model

The theoretical basis of this study is linguistic ecology, which is also called language ecology, ecology of language, or ecological linguistics [34]. The research content of ecologists and linguists is different in this interdisciplinary field [35], which is due to significant differences in researchers’ backgrounds in natural science and social science, respectively. The discipline of “linguistic ecology” is an extension of social science for linguists and involves sociolinguistics, functional linguistics, linguistic typology, and other sub-disciplines [36–40]. For ecologists, this discipline expands natural sciences and is concerned with environmental science, statistics, geography, biology, climatology, and other related disciplines [11, 41, 42]. In this study, linguistic ecology is primarily understood from the perspective of the natural sciences, and it is a new discipline with roots in human ecology.

**Fig 1:**
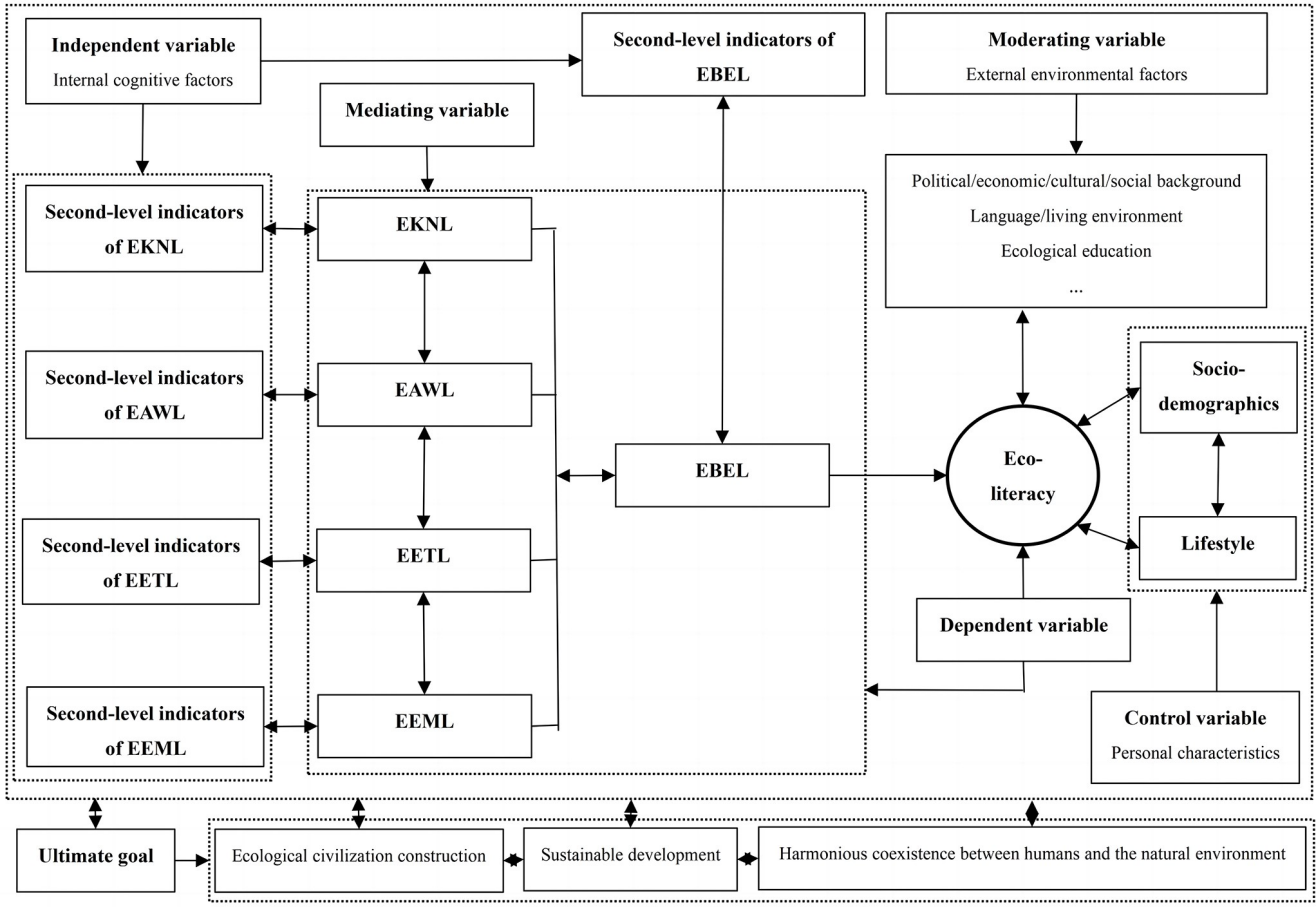
The underlying mechanism model of ecoliteracy in linguistic ecology. (Note: The details of second-level indicators are shown in Endnotes [11]).

In linguistic ecology, the internal indicators of ecoliteracy include five dimensions (EKNL, EAWL, EETL, EEML, and EBEL), among which EKNL is an important foundation, EAWL indicates the direction of action, EETL emphasizes moral standards, EEML is the internal driving force, and EBEL is the ultimate fundamental goal (see [11]). Under each dimension, four second-level indicators guide different aspects of the content in that dimension. They are simultaneously affected by many other surrounding factors (i.e., external environmental factors and personal characteristics factors). The underlying mechanism of ecoliteracy is summarized in Fig 1.

Fig 1 indicates that the underlying mechanism of ecoliteracy comprises five variables. The model systematically visualizes the formation and development of ecoliteracy, a dynamic and cyclical process.

The independent variable in this study referred to the internal cognitive factors of the inhabitants of Guiyang City, that is, the second-level indicators under the five dimensions of ecoliteracy. The cognitive factors of ecological knowledge, awareness, ethics, and emotions are at the base of the model. The participants’ mastery of these four aspects affected their corresponding ecoliteracy levels. These four dimensions of ecoliteracy will then affect the ecological behavior of inhabitants and produce specific EBEL. Under the combined effect of these five-dimensional levels (FDs) of ecoliteracy, the overall level of inhabitants’ ecoliteracy is formed.

The dependent variable in this study was ecoliteracy demonstrated by the inhabitants of Guiyang City.

The mediating variable includes FDs, which bridge the second-level indicators of ecoliteracy and the overall ecoliteracy (OEL) level and play an intermediary role. Moreover, for ecoliteracy as a dependent variable, FDs are independent variables.

The moderating variable (the moderator) in this study emphasized the external environmental factors that affect the ecoliteracy levels of the Guiyang inhabitants. These factors include political, economic, cultural, social, language, and educational environments. Therefore, they indirectly interfere with inhabitants’ levels of ecoliteracy.

The control variables in this study were personal characteristics, i.e., sociodemographic factors (gender, age, ethnicity, living area and type, educational background, current main identity, and family structure), as well as lifestyle (psychographics) of the participants (see the following section for details).

Inhabitants’ ecoliteracy characterization under the model reacts with the various internal cognitive factors of ecoliteracy and affects the level of ecoliteracy. Fundamentally, the ultimate goal of the model presented in this study is to promote China’s ecological civilization construction, sustainable development, and harmonious coexistence between humans and the natural environment. These factors also become external environmental factors that affect inhabitants’ ecoliteracy, cyclically driving its formation and improvement.

## Methods

### Data description

We designed an effective questionnaire for this study to explore ecoliteracy levels [11]. The study was approved by the Ethics Committee of the School of Foreign Studies, South China Agricultural University (200921). The questionnaire consisted of three parts, covering a total of 60 survey questions (including a self-assessment question on the respondents’ ecoliteracy level). The first part was related to sociodemographic characteristics (11 questions) [12]. The second part was in the form of a five-point Likert scale, which was used to examine the participants’ ecoliteracy levels (40 questions) with a score range (40–200) described elsewhere [11]. The third part considered participants’ attitudes toward ecological issues and their ecological behaviors (i.e., lifestyle characteristics; eight questions).

The second part was the core content of the questionnaire, which quantitatively evaluated the participants’ ecoliteracy levels. Specific questions were designed based on second-level indicators under the five dimensions of ecoliteracy, as shown in Fig 1. Each dimension included eight survey questions (score range: 8–40). After testing, the reliability (Cronbach’s alpha: 0.888) and validity (estimate: 0.67; CR: 0.95; AVE: 0.49) of the questionnaire were determined to be within reasonable ranges. To date, the first two parts of the survey have been completed [11, 12]. This study focused on the third section of the questionnaire, specifically as a combined study of the second and third parts. After understanding the ecoliteracy levels of participants with different lifestyle characteristics and analyzing the reasons for the results, strategies were proposed to improve low levels of ecoliteracy in participants.

Specifically, the lifestyle characteristics measured in the questionnaire referred to attitudes of importance to nature, participating in outdoor activities, and improving the level of ecoliteracy, as well as behavior in terms of frequency of daily outdoor activities, frequency of main activities in ecological areas, frequency of participation in volunteer activities related to ecological and environmental protection, and frequency of using ecological knowledge (S1 Questionnaire). The third part included another question to examine the main factors contributing to the participants’ ecoliteracy. This question was mainly designed for an in-depth exploration of ways to improve the level of ecoliteracy and was not directly related to the aim of this report. Therefore, this question was not analyzed or discussed. These seven lifestyles may contribute important benefits for individuals and society. They remind people to develop great living habits while protecting the eco-environment and promoting the sustainability of the community’s natural and social environment.

### Data collection and analysis

The survey collection process was completed by May 2021. The survey was undertaken by combining online participation and paper distribution, strictly limited to the population of the ten administrative regions of Guiyang City, and was randomly sampled at specific percentages. The subject of this study is all the permanent inhabitants of Guiyang, that is, the population who had lived in Guiyang City for more than half a year before the start of the investigation. We sent out 1,100 questionnaires, and 988 valid questionnaires were analyzed, constituting a survey ratio of 1:5000 of inhabitants of each administrative region of Guiyang City. The number of questionnaires exceeded the minimum sample size needed to achieve a significance level of α = 0.01 [12] and was based entirely on voluntary participation. Participants’ informed consent was obtained in the form of a multiple-choice question before the questionnaire began (fully anonymized), and they were allowed to discontinue the survey at any time. If the participants were minors, their informed consent and the answers to the questionnaire were assisted by their parents or guardians.

Microsoft Excel was used for data collation [11, 12] and the statistical software SPSS 25.0 was used for analysis. One-way analysis of variance (ANOVA) was performed as the main statistical method. Data on lifestyle characteristics were collated first. A one-way ANOVA was undertaken on ecoliteracy levels and lifestyle characteristics. Significant differences in OEL levels and FDs among Guiyang inhabitants with different lifestyle characteristics were determined at *p*<0.05. However, this difference was not statistically significant. Posthoc tests were also performed. However, due to space limitations, the results of the OEL only were used to consider the ecoliteracy levels of the participants from the overall representation. Finally, this article focused on analyzing lifestyle characteristics that showed significant differences, and data without statistical significance was not discussed. Intervention effects on inhabitants’ lifestyles were observed according to factors with significant differences.

## Results

According to the research questions raised in this article, after we have sorted out the underlying mechanism of ecoliteracy from the perspective of linguistic ecology, this section focuses on the results of combining different lifestyle characteristics with ecoliteracy. Through the data results, it can be seen what kind of lifestyle is beneficial to people’s ecoliteracy level. This also proves how the underlying mechanism of ecoliteracy works from another aspect.

### Attitude toward the importance of nature

**Fig 2:**
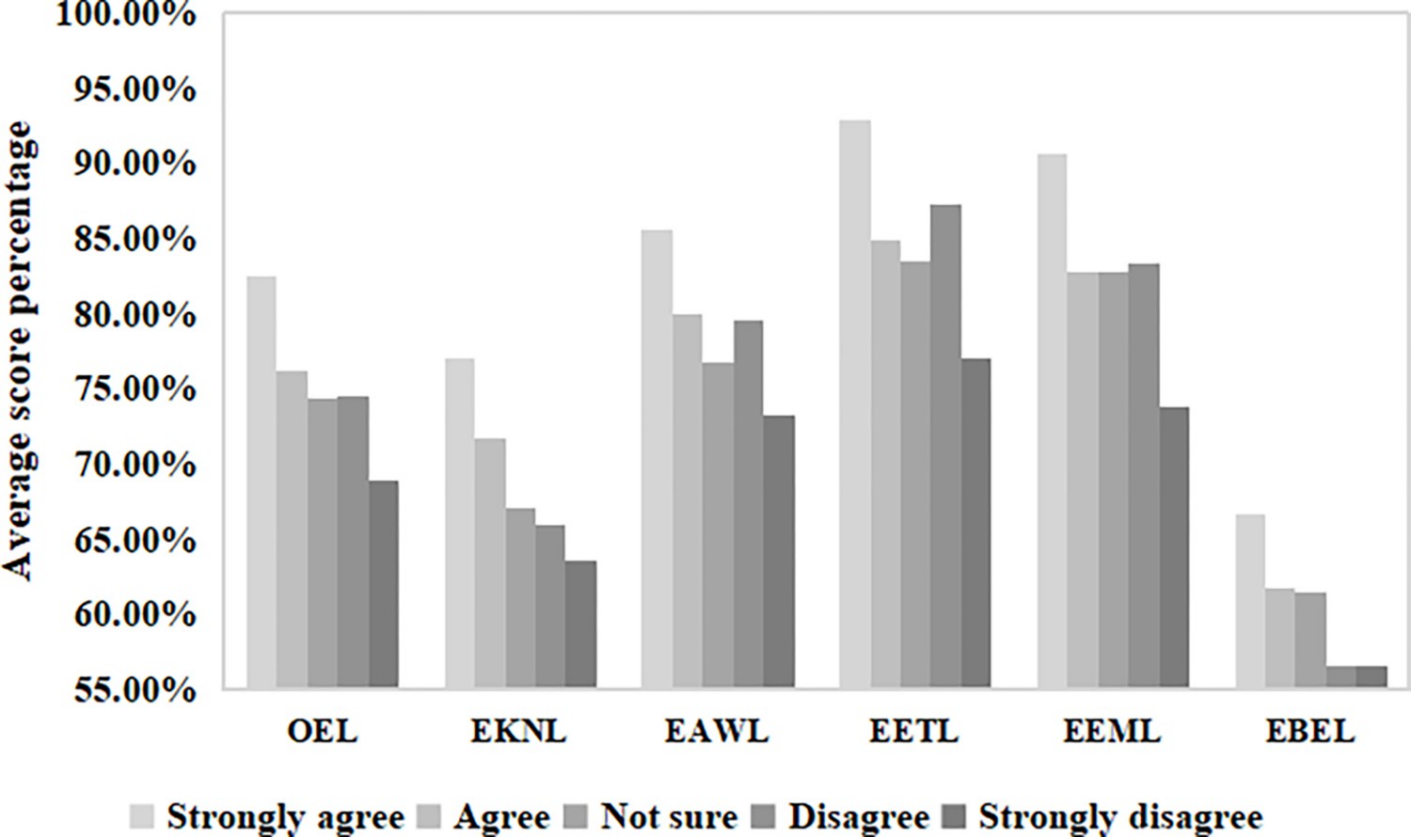
Average score percentages of attitudes toward the importance of nature and corresponding ecoliteracy levels.

The different attitudes of the Guiyang inhabitants toward nature reflected their different levels of ecoliteracy (Fig 2). The one-way ANOVA results show that the participants’ attitudes toward nature were significantly different regarding their OEL levels and FDs (Table 1). The significance coefficients of this factor were all *p* = 0.000. Inhabitants of Guiyang City who considered nature to be very important in their lives had the highest levels of OEL (165.12±13.973, 82.56%), EKNL (30.85±4.865, 77.13%), EAWL (34.22±3.707, 85.55%), EETL (37.14±3.046, 92.85%), EEML (36.24±3.263, 90.60%), and EBEL (26.67±5.271, 56.68%). With the decline in inhabitants’ attitudes toward nature, there was a slight upward trend in the level of ecoliteracy within five dimensions (OEL, EAWL, EETL, EEML, and EBEL). Overall, however, the level of ecoliteracy gradually declined with a decrease in positive attitudes toward nature.

**Table 1:** One-way ANOVA of the influence of attitudes toward the importance of nature on ecoliteracy levels.

	Attitude toward the importance of nature	Number	Mean	Standard deviation	F	*p*
OEL	Strongly agree	633	165.12	13.973	62.615	.000
	Agree	227	152.50	13.886		
	Not sure	75	148.71	17.432		
	Disagree	36	149.08	14.596		
	Strongly disagree	17	137.71	18.950		
EKNL	Strongly agree	633	30.85	4.865	23.633	.000
	Agree	227	28.67	4.994		
	Not sure	75	26.84	5.815		
	Disagree	36	26.39	5.608		
	Strongly disagree	17	25.41	6.226		
EAWL	Strongly agree	633	34.22	3.707	28.946	.000
	Agree	227	32.00	3.818		
	Not sure	75	30.73	4.726		
	Disagree	36	31.81	4.962		
	Strongly disagree	17	29.29	5.861		
EETL	Strongly agree	633	37.14	3.046	36.236^1^ 24.470^2^	.000^1^ .000^2^
	Agree	227	33.99	4.169		
	Not sure	75	33.39	5.798		
	Disagree	36	34.92	5.056		
	Strongly disagree	17	30.82	6.356		
EEML	Strongly agree	633	36.24	3.263	41.083^1^ 29.393^2^	.000^1^ .000^2^
	Agree	227	33.13	3.692		
	Not sure	75	33.15	5.080		
	Disagree	36	33.33	3.719		
	Strongly disagree	17	29.53	6.206		
EBEL	Strongly agree	633	26.67	5.271	13.725	.000
	Agree	227	24.71	4.908		
	Not sure	75	24.60	3.709		
	Disagree	36	22.64	3.498		
	Strongly disagree	17	22.65	4.271		

In further multiple comparisons, the results were as follows. The Guiyang inhabitants who considered nature to be very important in their lives (“strongly agree”) had significantly higher OEL levels than those who chose “agree” (*p* = 0.000), “not sure” (*p* = 0.000), “disagree” (*p* = 0.000), or “strongly disagree” (*p* = 0.000), with the average score differences greater than 10 points: 12.618, 16.409, 16.032, and 27.409, respectively. The OEL levels of inhabitants who believed that nature was completely unimportant in their lives (“strongly disagree”) were significantly lower than those of participants who selected “agree” (*p* = 0.000), “not sure” (*p* = 0.004), or “disagree” (*p* = 0.007), with average score differences higher than 10 points: 14.792, 11.001, and 11.377, respectively. In addition, participants who thought that nature was quite important in life (“agree”) scored significantly higher than those with the attitude of “not sure” (*p* = 0.048) on the OEL level, with an average score difference of 3.791.

### Attitude toward participating in outdoor activities

**Fig 3:**
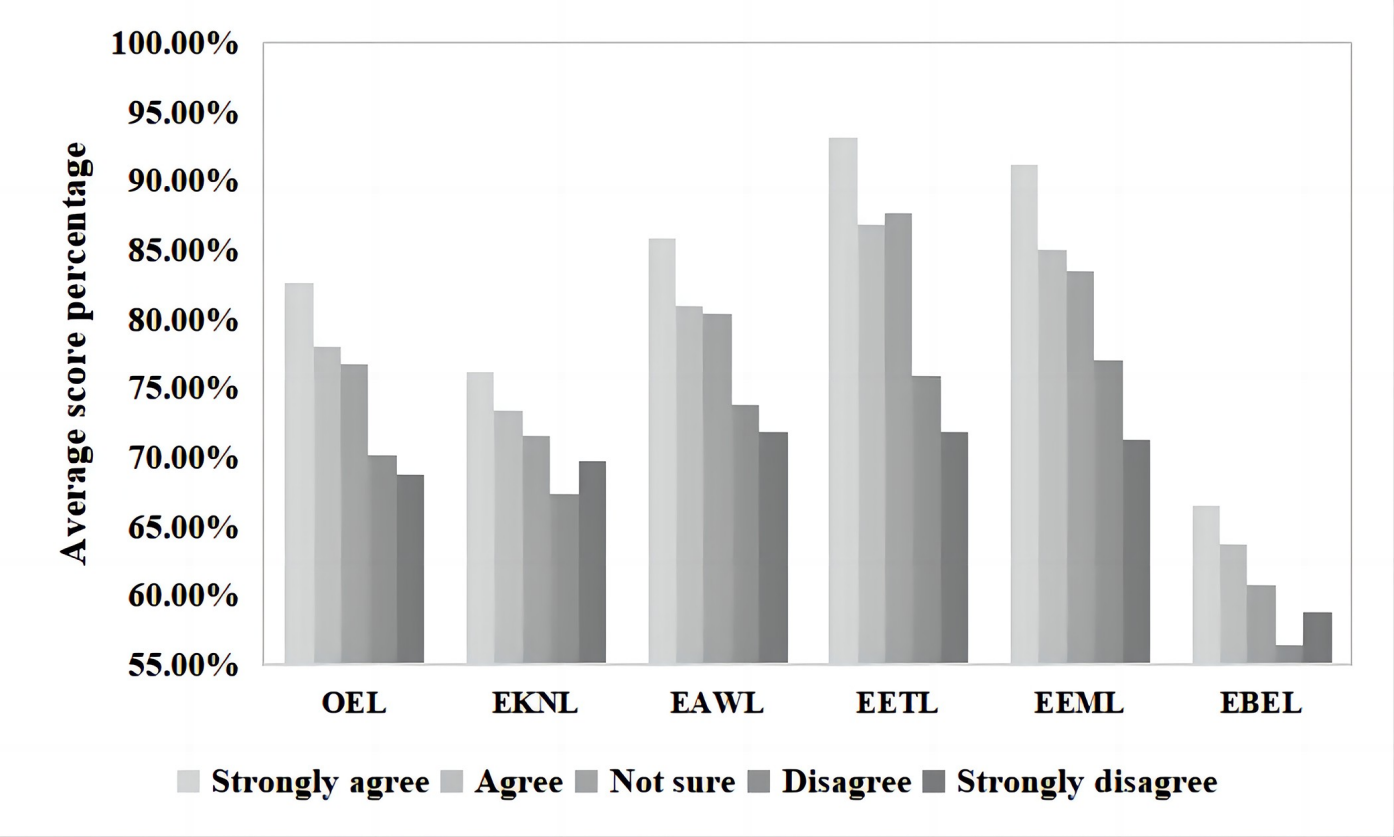
Average score percentages of attitudes toward outdoor activities and corresponding ecoliteracy levels.

The average score percentages of different attitudes toward participating in outdoor activities and the differences reflected in OEL levels and FDs are shown in Fig 3. The subsequent one-way ANOVA showed that the attitudes toward the importance of participating in outdoor activities greatly impacted all levels of ecoliteracy and that there were significant differences, with all coefficients at *p* = 0.000 (Table 2). Those who thought participating in outdoor activities was very important (“strongly agree”) had the highest average OEL level scores (165.23±15.095, 82.62%). In contrast, Guiyang inhabitants who believed that participating in outdoor activities was completely unimportant (“strongly disagree”) had the lowest average OEL level scores (137.45±26.086, 68.73%). Regarding FDs, participants who chose the option “strongly agree” had significantly higher ecoliteracy scores than those who chose one of the other four options, and they also had the highest levels of ecoliteracy in their corresponding dimensions.

**Table 2:** One-way ANOVA of the influence of attitudes toward outdoor activities on ecoliteracy levels.

	Attitude toward outdoor activities	Number	Mean	Standard deviation	F	*p*
OEL	Strongly agree	496	165.23	15.095	32.575^1^ 24.823^2^	.000^1^ .000^2^
	Agree	339	156.09	13.357		
	Not sure	127	153.65	16.708		
	Disagree	15	140.27	16.735		
	Strongly disagree	11	137.45	26.086		
EKNL	Strongly agree	496	30.48	5.441	5.761	.000
	Agree	339	29.39	4.715		
	Not sure	127	28.64	5.358		
	Disagree	15	26.93	5.311		
	Strongly disagree	11	27.91	6.316		
EAWL	Strongly agree	496	34.36	3.955	18.616^1^ 13.590^2^	.000^1^ .000^2^
	Agree	339	32.41	3.659		
	Not sure	127	32.18	4.255		
	Disagree	15	29.53	4.998		
	Strongly disagree	11	28.73	6.973		
EETL	Strongly agree	496	37.29	3.133	32.783^1^ 20.735^2^	.000^1^ .000^2^
	Agree	339	34.77	3.898		
	Not sure	127	35.10	4.968		
	Disagree	15	30.40	5.642		
	Strongly disagree	11	28.73	7.268		
EEML	Strongly agree	496	36.48	3.326	40.708^1^ 24.660^2^	.000^1^ .000^2^
	Agree	339	34.01	3.473		
	Not sure	127	33.41	4.638		
	Disagree	15	30.80	3.121		
	Strongly disagree	11	28.55	7.660		
EBEL	Strongly agree	496	26.62	5.541	8.240	.000
	Agree	339	25.51	4.584		
	Not sure	127	24.31	4.407		
	Disagree	15	22.60	4.808		
	Strongly disagree	11	23.55	4.591

*In the posthoc tests conducted on this dataset, the participants who held a very important attitude (“strongly agree”) toward outdoor activities were different in their OEL levels from those who chose “agree” (p = 0.000), “not sure” (p = 0.000), “disagree” (p = 0.000), or “strongly disagree” (p = 0.035). Among them, the difference between the average of participants who chose “strongly agree” and “agree” was the smallest (9.144), and the difference between “strongly agree” and “strongly disagree” was the largest, with a score difference of 27.775. In addition, the OEL levels of Guiyang inhabitants who held an “agree” attitude were significantly higher than those who held a “disagree” attitude (p = 0.019), with an average difference of 15.819*.

### Attitude toward improving the level of self-ecoliteracy

**Fig 4:**
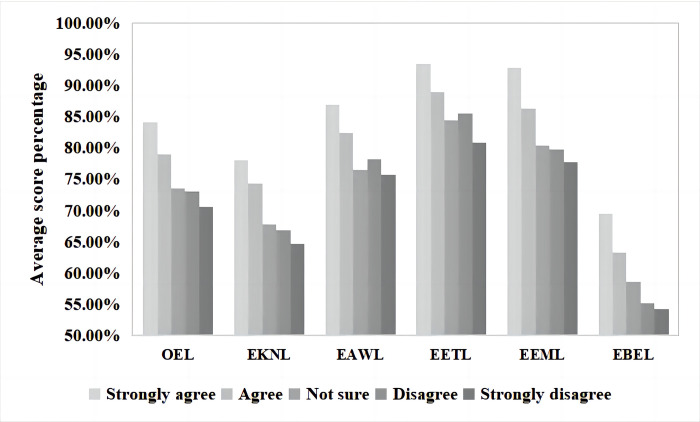
Average score percentages of self-interest in improving ecoliteracy level on actual ecoliteracy levels.

Differences in the levels of ecoliteracy among inhabitants with different levels of interest were subtle (Fig 4). The results of the one-way ANOVA showed that respondents with different interests in improving their ecoliteracy levels had significant differences in their OEL levels and FDs. The significance coefficients were all *p* = 0.000. Participants who were very interested in improving their ecoliteracy levels had the highest OEL level scores (168.38±15.138, 84.19%), while those who were not interested in improving their ecoliteracy levels at all had the lowest OEL level scores (141.33±9.722, 70.67%). The results are presented in Table 3.

**Table 3:** One-way ANOVA of the influence of participants’ interest in improving their ecoliteracy level on actual ecoliteracy levels.

	Improvement interest	Number	Mean	Standard deviation	F	*p*
OEL	Strongly agree	371	168.38	15.138	76.663	.000
	Agree	440	158.19	12.745		
	Not sure	116	147.16	15.546		
	Disagree	46	146.30	13.840		
	Strongly disagree	15	141.33	9.722		
EKNL	Strongly agree	371	31.21	5.371	21.668	.000
	Agree	440	29.74	4.555		
	Not sure	116	27.15	5.685		
	Disagree	46	26.78	5.116		
	Strongly disagree	15	25.87	4.794		
EAWL	Strongly agree	371	34.79	3.878	33.733	.000
	Agree	440	33.01	3.699		
	Not sure	116	30.59	4.540		
	Disagree	46	31.30	3.955		
	Strongly disagree	15	30.33	3.177		
EETL	Strongly agree	371	37.40	3.121	28.086	.000
	Agree	440	35.58	3.852		
	Not sure	116	33.81	5.357		
	Disagree	46	34.24	4.945		
	Strongly disagree	15	32.33	4.981		
EEML	Strongly agree	371	37.15	3.156	68.900	.000
	Agree	440	34.53	3.303		
	Not sure	116	32.20	4.851		
	Disagree	46	31.91	3.776		
	Strongly disagree	15	31.13	4.155		
EBEL	Strongly agree	371	27.84	5.594	34.125	.000
	Agree	440	25.34	4.347		
	Not sure	116	23.42	3.939		
	Disagree	46	22.07	4.711		
	Strongly disagree	15	21.67	4.499		

It can be seen from the posthoc test results that the inhabitants who were very interested in improving their ecoliteracy levels (“strongly agree”) had significantly higher scores than those who were somewhat interested (“agree,” *p* = 0.000), “not sure” (*p* = 0.000), not interested (“disagree,” *p* = 0.000), or not interested at all (“strongly disagree,” *p* = 0.000), with average score differences of 10.190, 21.219, 22.078, and 27.049, respectively. Those who were more interested (“agree”) in improving their ecoliteracy levels had significantly higher OEL levels than those who were “not sure” (*p* = 0.000), not very interested (“disagree”, *p* = 0.000), or not interested at all (“strongly disagree”, *p* = 0.000); the average score differences were 11.029, 11.889, and 16.860, respectively.

### Frequency of daily outdoor activity

**Fig 5:**
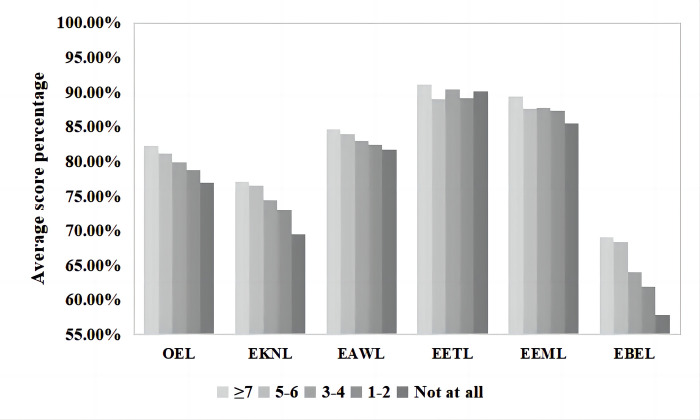
Average score percentages of outdoor activity frequency and ecoliteracy levels.

Behavioral characteristics represent a way of contact with the real world which can help train citizens to think and act ecologically and professionally [43]. In terms of OEL levels and FDs, the results of the one-way ANOVA (Table 4) show that there was no significant difference in the frequency of outdoor activities among the respondents corresponding to EAWL (*p* = 0.117), EETL (*p* = 0.231), and EEML (*p* = 0.066). However, OEL (*p* = 0.000), EKNL (*p* = 0.000), and EBEL (*p* = 0.000) differed significantly according to the frequency of outdoor activities. The average percentage scores of the results for each level are shown in Fig 5.

**Table 4:** One-way ANOVA of the influence of outdoor activity frequency on ecoliteracy levels.

	Outdoor activity frequency/Week	Number	Mean	Standard deviation	F	*p*
OEL	≥7	152	164.58	17.700	8.563	.000
	5–6	192	162.18	15.752		
	3–4	286	159.81	15.313		
	1–2	282	157.58	15.024		
	Not at all	76	153.92	16.647		
EKNL	≧7	152	30.84	5.396	6.389	.000
	5–6	192	30.63	5.286		
	3–4	286	29.76	5.204		
	1–2	282	29.22	4.970		
	Not at all	76	27.82	5.321		
EBEL	≧7	152	27.66	5.939	18.385	.000
	5–6	192	27.34	4.765		
	3–4	286	25.64	4.869		
	1–2	282	24.79	4.738		
	Not at all	76	23.13	4.588		

*Note: (1) EAWL: *p* = 0.117; (2) EETL: *p* = 0.231; (3) EEML: *p* = 0.066.

As shown in Table 4, the scores of the participants’ OEL levels gradually increased from the choice of “not at all” to engaging in outdoor activities more than seven times per week (≥7), which had the highest OEL levels (164.58±17.700, 82.29%), and participants who chose “not at all” had the lowest OEL levels, with an average score of only 153.92±16.647 (76.96%).

The posthoc test results showed respondents who engaged in outdoor activities seven times or more per week had significantly higher OEL levels than those who did so 3–4 times a week (*p* = 0.003), 1–2 times a week (*p* = 0.000), or not at all (*p* = 0.000), with average score differences of 4.771, 6.997, and 10.658, respectively. The inhabitants who engaged in outdoor activities 5–6 times a week showed significantly higher OEL levels than those of inhabitants who indicated 1–2 times (*p* = 0.002) or not at all (*p* = 0.000) with average differences in scores of 4.601 and 8.261, respectively. In addition, those who engaged in outdoor activities 3–4 times a week also had significantly higher OEL levels than inhabitants who chose “not at all” (*p* = 0.004), with a score difference of 5.887.

### Frequency of main activities in ecological areas

**Fig 6:**
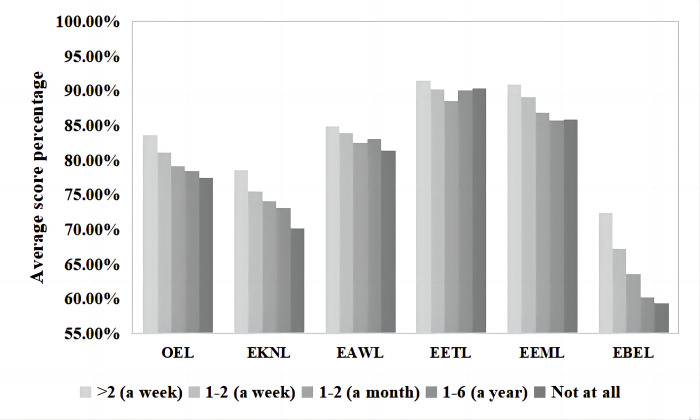
Average score percentages of activity frequency in ecological areas and corresponding ecoliteracy levels.

In terms of OEL levels and FDs for activities in ecological areas, the average score percentages for each dimension showed subtle differences among the frequencies (Fig 6). The results of the one-way ANOVA (Table 5) showed no significant difference in the activity frequencies in terms of their EAWL (*p* = 0.069) and EETL (*p* = 0.062). There were significant differences in OEL (*p* = 0.000), EKNL (*p* = 0.000), EEML (*p* = 0.000), and EBEL (*p* = 0.000). Those respondents who visited ecological areas more than twice a week had the highest OEL levels (167.30±17.769, 83.65%). Inhabitants who did not visit ecological areas (“not at all”) had the lowest OEL levels (154.89±16.779, 77.45%).

**Table 5:** One-way ANOVA of the influence of activity frequency in ecological areas on ecoliteracy levels.

	Activity frequency in ecological areas	Number	Mean	Standard deviation	F	*p*
OEL	>2 (a week)	128	167.30	17.769	13.676	.000
	1–2 (a week)	251	162.39	14.314		
	1–2 (a month)	320	158.29	15.644		
	1–6 (a year)	198	156F8.94	15.309		
	Not at all	91	154.89	16.779		
EKNL	>2 (a week)	128	31.42	5.876	6.543	.000
	1–2 (a week)	251	30.19	5.073		
	1–2 (a month)	320	29.62	4.999		
	1–6 (a year)	198	29.28	4.916		
	Not at all	91	28.09	5.723		
EEML	>2 (a week)	128	36.39	3.590	8.277	.000
	1–2 (a week)	251	35.65	3.461		
	1–2 (a month)	320	34.77	4.099		
	1–6 (a year)	198	34.29	4.137		
	Not at all	91	34.34	4.191		
EBEL	>2 (a week)	128	28.95	5.848	26.735	.000
	1–2 (a week)	251	26.89	4.912		
	1–2 (a month)	320	25.46	4.472		
	1–6 (a year)	198	24.10	4.499		
	Not at all	91	23.74	5.597		

*Note: (1) EAWL: *p* = 0.069; (2) EETL: *p* = 0.062.

According to the posthoc test results, the inhabitants of Guiyang City who visited ecological areas more than twice a week exhibited OEL levels significantly higher than those who visited these areas 1–2 times a week (*p* = 0.004), 1–2 times a month (*p* = 0.000), 1–6 times a year (*p* = 0.000), or almost never (“not at all”) (*p* = 0.000), with average score differences of 4.906, 9.009, 10.352, and 12.407, respectively. In addition, the OEL levels of citizens who were active in ecological areas 1–2 times a week were significantly higher than those of the inhabitants who visited them 1–2 times a month (*p* = 0.002), 1–6 times a year (*p* = 0.000), or almost never (“not at all”) (*p* = 0.000), with average score differences of 4.103, 5.446, and 7.500, respectively.

### Frequency of participating in volunteer activities

**Fig 7:**
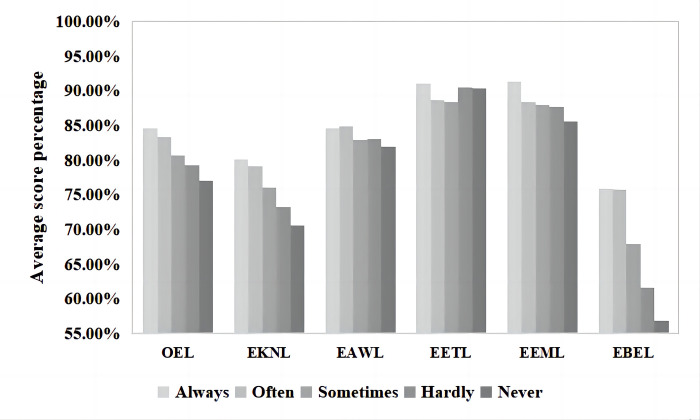
Average score percentages of participating in volunteer activities and corresponding ecoliteracy levels.

The volunteer activities discussed here only relate to protecting the eco-environment (see Fig 7 for preliminary statistics). Based on the survey results of the one-way ANOVA (Table 6), no significant difference was observed in EAWL (*p* = 0.137) and EETL (*p* = 0.075) levels. However, significant differences were observed in the levels of OEL (*p* = 0.000), EKNL (*p* = 0.000), EEML (*p* = 0.002), and EBEL (*p* = 0.000). Inhabitants who participated in volunteer activities had the highest OEL levels (169.24±17.576, 84.62%), and inhabitants who did not participate in volunteer activities at all (“never”) had the lowest OEL levels (154.20±15.594, 77.10%).

**Table 6:** One-way ANOVA of the influence of participating in volunteer activities on ecoliteracy levels.

	Participating in volunteer activities	Number	Mean	Standard deviation	F	*p*
OEL	Always	58	169.24	17.576	18.569	.000
	Often	112	166.84	17.728		
	Sometimes	226	161.38	16.995		
	Hardly	404	158.50	13.503		
	Never	188	154.20	15.594		
EKNL	Always	58	32.03	5.594	12.508	.000
	Often	112	31.66	5.637		
	Sometimes	226	30.43	4.963		
	Hardly	404	29.30	4.832		
	Never	188	28.25	5.485		
EEML	Always	58	36.53	3.803	4.347	.002
	Often	112	35.38	4.061		
	Sometimes	226	35.19	4.384		
	Hardly	404	35.08	3.660		
	Never	188	34.24	3.871		
EBEL	Always	58	30.38	6.445	76.531	.000
	Often	112	30.32	4.759		
	Sometimes	226	27.17	4.077		
	Hardly	404	24.65	3.966		
	Never	188	22.76	5.150		

*Note: (1) EAWL (*p* = 0.137); (2) EETL (*p* = 0.075).

In the posthoc tests, inhabitants who participated in volunteer activities often had significantly higher OEL levels than those who only participated “sometimes”(*p* = 0.001), “hardly ever” (*p* = 0.000), or “never” (*p* = 0.000), with relatively large differences: 7.856, 10.746, and 15.045, respectively. Inhabitants who often participated in volunteer activities also had significantly higher OEL levels than those who participated only “occasionally” (*p* = 0.002), “hardly ever” (*p* = 0.000), or “never” (*p* = 0.000), with average differences in scores of 5.454, 8.344, and 12.642, respectively. The levels were significantly higher for those participating in volunteer activities than for those who did not (*p* = 0.025) or never participated (*p* = 0.000). Finally, the OEL levels of inhabitants who “hardly ever” participated in volunteer activities were significantly higher than those of inhabitants who did not participate at all (“never”) (*p* = 0.002), with an average score difference of 4.298.

### Frequency of using ecological knowledge

**Fig 8:**
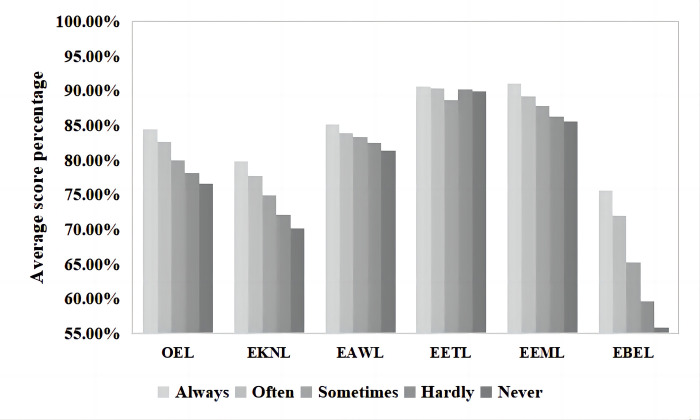
Average score percentages of ecological knowledge use and corresponding ecoliteracy levels.

The average score percentages of ecoliteracy levels corresponding to the different frequencies of ecological knowledge use are shown in Fig 8. One-way ANOVA was used to determine whether the different frequencies of the use of ecological knowledge by the inhabitants of Guiyang City had a significant effect on their OEL levels and FDs. The results (Table 7) show that there were no significant differences in the levels of EAWL (*p* = 0.107) and EETL (*p* = 0.266), based on the different frequencies of ecological knowledge use in study or work. However, significant differences were observed in the levels of OEL (*p* = 0.000), EKNL (*p* = 0.000), EEML (*p* = 0.000), and EBEL (*p* = 0.000). Those participants who always used ecological knowledge in their study or work had the highest OEL levels (168.97±19.576, 84.49%), while those who did not use ecological knowledge at all (“never”) had the lowest OEL levels (153.22±16.988, 76.61%).

**Table 7:** One-way ANOVA of the influence of ecological knowledge use on ecoliteracy levels.

	Ecological knowledge use	Number	Mean	Standard deviation	F	*p*
OEL	Always	88	168.97	19.576	21.270	.000
	Often	174	165.30	14.745		
	Sometimes	293	160.05	16.163		
	Hardly	348	156.45	13.429		
	Never	85	153.22	16.988		
EKNL	Always	88	31.92	6.060	11.858	.000
	Often	174	31.09	4.771		
	Sometimes	293	29.97	4.886		
	Hardly	348	28.87	5.152		
	Never	85	28.06	5.607		
EEML	Always	88	36.43	4.118	6.372	.000
	Often	174	35.71	3.509		
	Sometimes	293	35.14	4.229		
	Hardly	348	34.54	3.650		
	Never	85	34.25	4.375		
EBEL	Always	88	30.27	6.289	67.113	.000
	Often	174	28.79	3.954		
	Sometimes	293	26.10	4.353		
	Hardly	348	23.89	4.394		
	Never	85	22.36	5.052		

*Note: (1) EAWL: *p* = 0.107; (2) EETL: *p* = 0.266.

In the posthoc tests, the OEL levels of Guiyang inhabitants who “always” used ecological knowledge in their study or work were significantly higher than those who chose “sometimes” (*p* = 0.000), “hardly ever” (*p* = 0.000), or “never” (*p* = 0.000). The average differences in scores were 8.911, 12.518, and 15.742, respectively. Inhabitants who often used ecological knowledge had significantly higher OEL levels than those who only “sometimes” used it (*p* = 0.000), “hardly ever” used it (*p* = 0.000), and “never” used it (*p* = 0.000), with differences between the average values of 5.250, 8.856, and 12.081, respectively. Respondents who used ecological knowledge only “sometimes” had significantly higher OEL levels than those who used ecological knowledge “rarely ever” (*p* = 0.003) or “never” (*p* = 0.000), with average differences in scores of 3.606 and 6.831, respectively.

## Discussion

This section is a sequential discussion of the data results of this study. The aim is to reveal the meaning behind the data and the pathways to improve the ecoliteracy level for different individuals or groups, thereby broadening the scope of application as an interdisciplinary content of ecoliteracy and linguistic ecology.

### Attitude toward the importance of nature

Guiyang inhabitants’ attitudes toward nature directly reflect their impression of nature, which affect their levels of ecoliteracy, particularly EAWL. Inhabitants who believe nature is highly important have strong ecological awareness and affection for nature. It further stimulates their desire to gain ecological knowledge, using such scientific knowledge to strengthen their levels of ecoliteracy and then using their ecological ethics to restrain themselves, as reflected in ecological behavior.

As for the effect of attitude toward the importance of nature on the corresponding level of ecoliteracy, there are many cases consistent with the results of this study[32, 44], whether in the consciousness of most people or in relevant researches. The implications of relevant research are as follows: people’s perceptions of nature will change over time, as will their attitudes toward nature [32, 45]. This study found that Guiyang inhabitants’ attitudes toward nature are influenced by various factors, such as their educational background, family influence, and the media. Therefore, deepening their understanding and internalization of the concept of ecological sustainability and reflecting on their current ecologically unsustainable behaviors can enhance their levels of ecoliteracy.

When ecological problems become increasingly prominent, the role of nature in people’s lives has become increasingly significant. This means that we have to focus on the inhabitants of Guiyang City, who are currently unconcerned with nature. This study can infer that most inhabitants of Guiyang City believe that nature is “very important” (“strongly agree”) or “relatively important” (“agree”) (n = 860), accounting for 87.04% of all participants. On the other hand, the proportion of those respondents who think nature is “unimportant” (“disagree”) or “completely unimportant” (“strongly disagree”) (n = 53) was only 5.36%. Therefore, when ecoliteracy is raised among inhabitants who consider nature unimportant, the influence of surrounding people is critical. Through interpersonal communication and joint work, they can indirectly influence low-level ecoliteracy inhabitants to reflect on their attitudes toward nature. This can result in higher levels of ecoliteracy.

### Attitude toward participating in outdoor activities

The inhabitants of Guiyang City who have a very positive attitude toward outdoor activities may not necessarily participate in those activities frequently, but do express the intention to participate. Such a tendency can directly affect their ecological awareness, emotional reactions, and propose more restrictive conduct through ecological ethics and therefore develop a higher level of EEML. These aspects are sufficient to motivate them to gain a higher level of ecoliteracy. Otherwise, inhabitants with a negative attitude about participating in outdoor activities will not have enough love for the outdoors, including ecology and nature. Therefore, this part of the population shows a lower level of ecoliteracy. The results of this section support the views of Sebba, Pitman et al. and others to varying degrees [32, 46].

It was also found that the number of participants who found outdoor activities unimportant or completely unimportant was very small (n = 26), accounting for only 2.63% of all participants. This percentage was sufficient to show that overall, the inhabitants of Guiyang City had a very positive attitude toward participating in outdoor activities, which is the first step in engaging in outdoor activities, which, in turn, will have an effect on positive ecological behavior. As a result, only three sets of data showed significant differences in the posthoc test of both levels of EKNL and EAWL. The most direct reason for this was that attitude toward outdoor activities reflected the level of thinking or awareness. At present, the publicity and education about ecology in Guiyang City are considered positive, and they strive to instill awareness and the helping of others. Therefore, in addition to consciously going outside into nature, people must influence others to jointly enhance a connection with nature and improve ecoliteracy levels [47]. However, the small difference in EKNL levels was due to this factor not being directly related to the level of ecological knowledge. Thus, strengthening the guidance of ecological awareness, ethics, emotions, and behaviors for those who are resistant to participating in outdoor activities and subtly improving their attitudes toward participating in outdoor activities is essential. This is an advantageous step in improving ecoliteracy levels.

### Attitude toward improving level of self-ecoliteracy

Those inhabitants of Guiyang City who were very interested (“strongly agree”) and quite interested (“agree”) in improving their ecological knowledge and understanding, as well as their level of ecoliteracy, had the highest levels of ecoliteracy, which were significantly higher than those of the other three levels of interest. This showed that interest in a particular field was relevant to the effect of knowledge and the cultivation of ability within that field. Tobias also pointed out that people interested in a subject will acquire more knowledge about it because they actively spend time on activities in that subject [48]. Over time, interest in this area has motivated people to form inherently stable literacy. Based on the results of this research, a high level of ecoliteracy was formed. In contrast, those Guiyang inhabitants who were less interested (“disagree” and “strongly disagree”) in ecological knowledge, understanding, and their ecoliteracy levels, had very low levels of ecoliteracy. However, the number of inhabitants in this group was small (n = 61), accounting for only 6.17% of the total participants. In summary, most inhabitants of Guiyang City (an ecologically advanced city) could manage their ecoliteracy level. Their good self-management has achieved the steady development of Guiyang’s ecologically-aware civilization.

Whether the ecoliteracy countermeasures proposed in each part of this research can be successfully realized depends on the interests of the inhabitants. This section further validates some of the discussion by Pitman et al., and Lin and Cai [32, 49]. The results showed that the proportion of very interested people (“strongly agree”) and quite interested (“agree”) in improving their ecological knowledge and understanding, and ecoliteracy levels reached 82.09% (n = 811). This suggests that many strategies proposed by this study, after such comparison of differences, are likely to be realized. Therefore, when we strive to implement measures for improving inhabitants with low ecoliteracy, attention needs to be given to those who are not very interested in their own ecoliteracy. Other measures can be more effectively implemented when their interests are successfully cultivated.

### Frequency of daily outdoor activity

In terms of daily outdoor activity, the ecoliteracy difference was specifically evident in ecological knowledge and behavior. Those inhabitants usually deeply understood the eco-environment because they had more ecological knowledge and took the initiative to visit nature areas to participate in activities, or they devoted themselves to nature just for exercise. Regardless of the reasons, they directly touched the sky, earth, flowers, trees, various animals in nature, and rocks, in contrast to viewing such things through windows or literature. Impressions of ecological knowledge are more profound when ecosystems are visited. Therefore, respondents who often participate in outdoor activities differed from other inhabitants with respect to levels of EKNL. The increase in the frequency of daily outdoor activities improved not only the inhabitants’ inherent EKNL, but also the use of their ecological knowledge to think critically about ecological issues because of what they saw and heard outdoors. This caused their EBEL to become significantly higher than that of other inhabitants. Under the combined effect of “knowledge” and “action,” the differences in the frequency of daily outdoor activities can produce significant differences in their ecoliteracy levels.

The research results here support the viewpoint of several researchers, such as McDaniel and Alley, Pitman et al. [33, 50]. They generally agree that with the increase in the frequency of daily outdoor activity, the level of ecoliteracy will be improved, especially the effect on the grasp of ecological knowledge and ecological action practice is relatively significant. For this characteristic behavioral factor, countermeasures can be found from two perspectives (the inhabitants and ecoliteracy), but the countermeasures are both aimed at those respondents who participate in outdoor activities less frequently and encourage those inhabitants to participate outside.

From their perspective, physical health is important and a prerequisite and foundation for successful study or work. This makes it easier to guide them toward participating in outdoor activities. In recent years, the nationwide physical fitness campaign has gradually been promoted and will indirectly influence the outdoor activity participation frequency of Guiyang inhabitants. From the perspective of ecoliteracy itself, special attention needs to be paid to learning ecological knowledge and the practice of ecological behavior of these inhabitants. We also need to inspire outdoor activity participants to include those who usually do not participate in. Then, the inhabitants with low frequency of daily outdoor activity are initiated to participate in outdoor activity and learn about ecology. This is more effective than learning about ecology through education alone. Ecological knowledge implemented through ecologically-based actions will effectively improve the ecoliteracy levels of inhabitants.

### Frequency of main activities in ecological areas

The landscape presented by the ecological area was more concentrated, and inhabitants did not need to seek nature. Local signage can allow information about plants, for example, to be provided and thus produce a certain level of understanding and knowledge of ecology from simple attendance. This may improve levels of EKNL. In terms of EEML, inhabitants willing to take the initiative to enter an ecological area for activities have a certain understanding of the ecology and environment, and want to discover further. Therefore, inhabitants who frequently visit ecological areas have higher EEML levels than those who do not. The comprehensive effect of these types of literacy on the behaviors of citizens leads to significant differences in their levels of EBEL. Ultimately, inhabitants often visiting ecological areas have a higher level of ecoliteracy, while inhabitants being less active in ecological areas have a relatively low level of ecoliteracy.

The views in this article are similar to those of Hammarsten et al. and Wells et al. [51, 52]. Among them, Wells et al. advocated learning plant science knowledge, cultivating interest in plants and improving the ecoliteracy of participants through participation in horticultural activities. Optimization of management and citizens should be prioritized to improve ecoliteracy levels in the process of ecological area activities.

On the one hand, managers must ensure the comprehensive and accurate introduction of different species in ecoregions so that more inhabitants are willing to visit. Managers in ecological areas also need to have a higher level of ecoliteracy and be able to continuously broaden their ecological knowledge to further improve their ecoliteracy levels. On the other hand, we still need to pay attention to the individuals of Guiyang inhabitants. Levels of ecoliteracy may not directly be improved when activities in ecological areas are offered; however, activities in ecological areas will reduce the pressure on citizens, cultivate an appreciation of nature, and so indirectly improve ecological knowledge of the area, and thus ecoliteracy. Therefore, this study advocates that the inhabitants of Guiyang City undertake activities in ecological areas after their daily study or work, not only to improve their physical and mental health but also for their ecoliteracy levels.

### Frequency of participating in volunteer activities

Volunteer experiences related to the ecology and environment can significantly affect the levels of ecoliteracy among Guiyang inhabitants, which is mainly reflected in their ecological knowledge, emotions, and behaviors. Participating in volunteer activities related to ecological and environmental protection is an active behavior of inhabitants and a manifestation of EBEL. These volunteers hoped to help complete the activities through their ecoliteracy, while wanting to deliver ecological content to the people they served. Citizens who want to participate in such volunteer activities also strongly appreciate and respect the eco-environment and have a high level of EEML. In terms of EKNL, by participating in volunteer activities for ecological and environmental protection, they will acquire a certain amount of ecological knowledge during the training and activities before such events, which not only improves the service ability of the volunteers but also enhances their EKNL levels. When their overall levels of ecoliteracy are improved, their willingness and ability to continue participating in volunteer activities related to ecological and environmental protection are also enhanced.

The research perspective that affirms the participation of related volunteer activities to improve people’s ecoliteracy level and protect the well-being of human survival has been verified [53, 54]. In this part of the study, the number of Guiyang inhabitants who always or often chose to participate in volunteer activities related to ecological and environmental protection was relatively small (n = 170), accounting for only 17.21% of all participants. In contrast, 59.92% (n = 592) of the participants reported that they “hardly ever” or “never” participated in volunteer activities related to ecological and environmental protection. This gap was proportionally very wide; more than half of Guiyang inhabitants rarely participated in volunteer activities related to ecological and environmental protection, and such activities could significantly affect their ecoliteracy levels. This requires the cultivation of inhabitants’ service awareness and encourages them to actively participate in ecological and environmental protection services to improve their levels of ecoliteracy. Increasing citizen participation in volunteer activities in the eco-environment will improve the ecoliteracy level effectively and quickly.

### Frequency of using ecological knowledge

The frequency of using ecological knowledge is directly related to a person’s level of EKNL, which is also explained by repeated training in a certain subject as an indispensable part of mastering a skill. Therefore, the inhabitants of Guiyang City who used ecological knowledge more frequently in their studies or work had higher levels of EKNL than those who did not. This fosters a significant appreciation for the eco-environment, gradually forming the EEML discussed in this study. Furthermore, inhabitants who improve their levels of EKNL and EEML through this approach will have ecological behaviors that are superior to others; i.e., they use their good ecological knowledge and emotions in their learning or working behaviors so that their overall levels of ecoliteracy are higher than those of inhabitants who use ecological knowledge less frequently. In other words, ecological knowledge’s important role in forming ecoliteracy is demonstrated again [11, 25].

Ecological knowledge is one of the most basic and important factors in the development of ecoliteracy. At present, the inhabitants of Guiyang City who always or often used ecological knowledge in their studies or work only accounted for 26.52% of the sample (n = 262), while inhabitants who rarely (“hardly ever”) or never use ecological knowledge accounted for 43.83% (n = 433), i.e., nearly half of the local inhabitants rarely use ecological knowledge in their studies or work. However, some learning or working content is not related to ecological knowledge; therefore, a question would be raised: how do people use ecological knowledge in their ordinary life? From the perspective of linguistic ecology, the finding of countermeasures can be focused on ecological discourse for situations in which the content of study or work has low relevance to the eco-environment. That is, in the act of their study or work, they should consciously use eco-beneficial discourses and control their wasteful behavior through ecological actions. Such inhabitants should gradually form strong ecological emotions and improve their ecoliteracy.

## Conclusions

Ecoliteracy is a key factor in achieving sustainable development in human society, and its role always exists in the harmonious relationship between humans and nature [32]. Currently, research on ecoliteracy issues is mainly concentrated within the discipline of ecology (e.g., [20]), and it is quite rare to use an interdisciplinary perspective for analysis, particularly the perspective of linguistic ecology (e.g., [11]). Therefore, this research on ecoliteracy is significant to the development planning of Guiyang City, the tenth most ecologically advanced city in China, and it is also significant to other cities in China and other countries. Based on constructing an ecoliteracy mechanism model, this article has analyzed and discussed a series of lifestyle characteristics factors and has determined three main conclusions.

First, from the perspective of linguistic ecology, the formation and development of ecoliteracy are carried out under dynamic and circular models. The coordination of five variables is required, including the independent variable (second-level indicators), mediating variable (FDs), moderating variable (external environmental factors), dependent variable (ecoliteracy), and control variable (personal characteristics) for improving the ecoliteracy and cycle of conscience of Guiyang inhabitants. Ultimately, an ecosystem can be built where humans and nature live harmoniously.

Second, this study considers the various variables in the ecoliteracy mechanism model but the focus has been on lifestyle characteristics (control variables). The study found that the seven characteristic lifestyle factors investigated here led to differences in the frequency of the participants’ activities, with significant differences in their OEL levels and, to varying degrees, in their FDs. This means that both the attitudes of Guiyang inhabitants toward ecological issues and their practice of ecological activities had strong positive effects on their ecoliteracy.

Third, improvements in inhabitants’ ecoliteracy can also promote changes in physical literacy. It is beneficial for enhancing the health and well-being of future generations [55]. Based on the results of the lifestyle intervention factors in this study, Guiyang inhabitants are encouraged to first maintain a positive attitude toward nature and then participate in outdoor activities and manage their ecoliteracy. On a personal level, such an attitude allows people to take the initiative to step into nature to strengthen their ecoliteracy and undertake physical exercise such as hiking, mountain climbing, or visiting forest parks. Not only does this improve health and reduce work- and life-related stress, but it also promotes ecoliteracy.

Although this study is significant for the sustainable development of society, it has a few limitations that should be further explored. Currently, the core of this study is "ecoliteracy", which is placed under the framework of linguistic ecology to explore the interaction between lifestyle interventions and ecoliteracy, but the realization of sustainable development goals has not been discussed. In subsequent studies, we can continue to apply the study of lifestyle interventions and ecoliteracy into the broader context of sustainable development and discuss its effectiveness on the realization of SDGs, such as the intrinsic value in Quality Education (SDG 4), Sustainable Cities and Communities (SDG 11) and Responsible Consumption and Production (SDG 12).

## Endnotes

1. In our study, before the one-way ANOVA, the data were tested for homogeneity of variance. If the homogeneity of variance had *p*<0.05, and the ratio of the maximum-to-minimum-variance of the factor was greater than 3, this factor needed to use a robust test method for mean equality to examine significant differences and trends. The two rows of values in F and *p* in each Table were evaluated by this method. The superscript “1” designates the F-value and significance of the Welch test. The superscript “2” designates the F-value and significance of the Brown-Forsythe test.
2. EKNL: Ecosystem knowledge; Knowledge of damage to the eco-environment; Knowledge of the relationship between humans and nature; Ecological and environmental protection knowledge.

EAWL: Ecological and environmental protection behavior subject consciousness; Ecological and environmental protection value awareness; Awareness of the severity of current ecological and environmental problems; Making judgments on the ecological and environmental damage encountered.

EETL: Correctly recognizing the relationship between humans and nature; The ethics and morality of protecting the eco-environment; Affirming the role of nature; Respecting and cherishing all living things.

EEML: Awe of the natural environment; Love for the natural environment; Sensitivity to natural environment protection; Ability to take responsibility for ecological and environmental issues.

EBEL: Daily practice of environmental protection; Participation in environmental education activities; Scientific environmental protection skills and methods; Positive influence on the environmental protection behavior of others.

## Supporting information

S1 Data (XLSX)

S1 File (ZIP)

S2 File (DOCX)

## References

[1] Xie M, Irfan M, Razzaq A, Dagar V. Forest and mineral volatility and economic performance: evidence from frequency domain causality approach for global data. Resources Policy. 2022; 76, 102685.

[2] Ishrakieh LM, Dagher L, Hariri SE. A financial stress index for a highly dollarized developing country: the case of Lebanon. Central Bank Review. 2020; 20, 43–52.

[3] Zhang CH, Khan I, Dagar V, Saeed A, Zafar MW. Environmental impact of information and communication technology: unveiling the role of education in developing countries. Technological Forecasting & Social Change. 2022; 178, 121570.

[4] Belaid F, Dagher L, Filis G. Revisiting the resource curse in the MENA region. Resources Policy. 2021; 73, 102225.

[5] Tang C, Irfan M, Razzaq A, Dagar V. Natural resources and financial development: role of business regulations in testing the resource-curse hypothesis in ASEAN countries. Resources Policy. 2022; 76, 102612.

[6] Dagher L, Hasanov FJ. Oil market shocks and financial instability in Asian countries. International Review of Economics and Finance. 2023; 84, 182–195.

[7] Urom C, Guesmi K, Abid I, Dagher L. Dynamic integration and transmission channels among interest rates and oil price shocks. The Quarterly Review of Economics and Finance. 2023; 87, 296–317.

[8] Bird L, Kreycik C, Friedman B. Green power marketing in the United States: a status report, 11th ed.; National Renewable Energy Laboratory. NREL/TP-6A2-44094, Golden, Colorado, USA; 2008.

[9] Dagar V, Khan MK, Alvarado R, Usman M, Zakari A, Rehman A, et al. Variations in technical efficiency of farmers with distinct land size across agro-climatic zones: evidence from India. Journal of Cleaner Production. 2021; 315, 128109.

[10] Irfan M, Elavarasan RM, Ahmad M, Mohsin M, Dagar V, Hao Y. Prioritizing and overcoming biomass energy barriers: application of AHP and G-TOPSIS approaches. Technological Forecasting & Social Change. 2022; 177, 121524.

[11] Ha CC, Huang GW, Zhang JE, Dong SM. Assessing ecological literacy and its application based on linguistic ecology: a case study of Guiyang City, China. Environmental Science and Pollution Research. 2022; 29 (13), 18741–18754. doi: 10.1007/s11356-021-16753-7 34704227 PMC8547900

[12] Ha CC, Dong SM. Identifying the most ecoliterate inhabitants in a top-ten ecologically advanced city of China: a sociodemographic perspective. Sustainability. 2023; 15 (4), 3054.

[13] Stibbe A. The Handbook of Sustainability Literacy: Skill for a Changing World. Green Books, Dartington; 2009.

[14] McBride BB, Brewer CA, Berkowitz AR, Borrie WT. Environmental literacy, ecological literacy, ecoliteracy: what do we mean and how did we get here?. Ecosphere. 2013; 4 (5), 1–20.

[15] Huang GW, Ha CC. The relationship between ecological literacy and ecolinguistics. Foreign Language Education. 2021; 42 (1), 15–19. (In Chinese)

[16] Casper AMA, Balgopal MM. Conceptual change in natural resource management students’ ecological literacy. Environmental Education Research. 2017. 10.1080/13504622.2017.1350830.

[17] Orr DW. Ecological Literacy: Education and the Transition to a Postmodern World. State University of New York Press, Albany, USA; 1992.

[18] Berkowitz A, Brewer C, McBride B. Essential elements of ecological literacy and the pathways needed for all citizens to achieve it. 93rd Ecological Society of America Annual Meeting. Symposium 12, August 3–8, 2008. http://eco.confex.com/eco/2008/techprogram/P9585.HTM.

[19] Balgopal MM, Wallace AM. Decisions and dilemmas: using writing to learn activities to increase ecological literacy. The Journal of Environmental Education. 2009; 40 (3), 13–26.

[20] Pitman SD, Daniels CB. Quantifying ecological literacy in an adult western community: the development and application of a new assessment tool and community standard. PLoS One. 2016; 11(3), e0150648, 1–18. doi: 10.1371/journal.pone.0150648 26938258 PMC4777481

[21] Casper AMA, Fernández-Giménez ME, Balgopal MM. A tool for measuring ecological literacy: coupled human-ecosystem interactions. The Journal of Agricultural Education and Extension. 2020. 10.1080/1389224X.2020.1780139.

[22] Pitman SD, Daniels CB, Sutton PC. Ecological literacy and socio-demographics: who are the most eco-literate in our community, and why?. International Journal of Sustainable Development and World Ecology. 2016; 25 (1), 9–22.

[23] Arcury TA. Environmental attitude and environmental knowledge. Human Organization. 1990; 49 (4), 300–304.

[24] Morrone M, Mancl K, Carr K. Development of a metric to test group differences in ecological knowledge as one component of environmental literacy. The Journal of Environmental Education. 2001; 32 (4), 33–42.

[25] Coyle K. Ecological literacy in America; what ten years of NEETF/Roper Research and related studies say about environmental literacy in the U. S. National Environmental Education and Training Foundation. 2005. http://www.neefusa.org.

[26] Capra F, Stone MK. Smart by nature: schooling for sustainability. The Journal of Sustainability Education. 2010. http://www.jsedimensions.org/wordpress/tags/smart-by-nature/.

[27] Risser PG. Ecological literacy. Bulletin of the Ecological Society of America. 1986; 67 (4), 264–270.

[28] Capra F. The Hidden Connections: A Science for Sustainable Living. Random House Digital, Inc, New York, NY; 2004.

[29] Pickett STA, Cadenasso ML, Grove JM, Boone CG Groffman PM, Irwin E et al. Urban ecological systems: scientific foundations and a decade of progress. Journal of Environmental Management. 2011; 92 (3), 331–362. doi: 10.1016/j.jenvman.2010.08.022 20965643

[30] Davidson MF. Ecological literacy evaluation of the University of Iceland faculty, staff, and students: implications for a university sustainability policy. Unpublished Ph.D Thesis, University of Iceland, Reykjavik. 2010. http://s3.amazonaws.com/zanran_storage/skemman.is/ContentPages/115928815.pdf.

[31] Pitman SD, Daniels CB. Understanding how nature works: five pathways towards a more ecologically literate world—a perspective. Austral Ecology. 2020; 1–10.

[32] Pitman SD, Daniels CB, Sutton PC. Ecological literacy and psychographics: lifestyle contributors to ecological knowledge and understanding. International Journal of Sustainable Development and World Ecology. 2017; 1–14. 10.1080/13504509.2017.1333047.

[33] Pitman SD, Daniels CB, Sutton PC. Characteristics associated with high and low levels of ecological literacy in a western society. International Journal of Sustainable Development and World Ecology. 2018; 25 (3), 227–237. 10.1080/13504509.2017.1384412.

[34] Huang GW. The emergence and development of ecolinguistics. Foreign Languages in China. 2016; 13 (1), 9–12. (In Chinese)

[35] Huang GW, Li WB. Ecolinguistics as applied linguistics. Modern Foreign Languages. 2021; 44 (5), 592–601. (In Chinese)

[36] Haugen E. The ecology of language. In Dil A.S. (ed.). The Ecology of Language: Essays by Einar Haugen. Stanford University Press, Stanford, 1972; pp. 325–339.

[37] Halliday MAK. New ways of meaning: the challenge to applied linguistics. J. Appl. 1990, (6), 7–16. Reprinted in Webster, J. (ed.). On Language and Linguistics, Vol. 3 in The Collected Works of MAK. Halliday. Continuum: London, UK, 2003; pp. 139–174.

[38] Garner M. Language: An Ecological View. Peter Lang, Bern; 2004.

[39] Alexander R, Stibbe A. From the analysis of ecological discourse to the ecological analysis of discourse. Language Sciences. 2014; (41), 104–110.

[40] Stibbe A. Ecolinguistics: Language, Ecology and the Stories We Live By, 2nd ed.; Routledge, London, UK; 2021.

[41] Zhang L, Huang GW, Li YT, Bao ST. The application of landseses in language carriers. International Journal of Sustainable Development and World Ecology. 2021a. 10.1080/13504509.2021.1920062.

[42] Zhang L, Huang GW, Li YT, Bao ST. A psychological perception mechanism and factor analysis in landsenses ecology: a case study of low-carbon harmonious discourse. International Journal of Environmental Research and Public Health. 2021b. doi: 10.3390/ijerph18136914 34203161 PMC8296938

[43] Nordlund LM. Teaching ecology at university—Inspiration for change. Glob. Ecol. Conserv. 2016; (7), 174–182. 10.1016/j.gecco.2016.06.008.

[44] Gu X, Huang BY, Wu JP. Family member’ attitudes toward nature-based activities and children’s environmental behaviors: the chain-mediating role of children’s frequency of contact with nature and connection to nature. China Journal of Health Psychology. 2022; 30 (1), 80–85. (In Chinese)

[45] Haraway DJ. Simians, Cyborgs, and Women: The Reinvention of Nature. Routledge, New York; 2013.

[46] Sebba R. The landscapes of childhood: the reflection of children’s attitudes. Environment and Behaviour. 1991; 23, 395–422.

[47] Oh RRY, Fielding KS, Nghiem LTP, Chang CC, Carrasco LR, Fuller RA. Connection to nature is predicted by family values, social norms and personal experiences of nature. Global Ecology and Conservation. 2021; (28), e01632. 10.1016/j.gecco.2021.e01632.

[48] Interest Tobias S., prior knowledge, and learning. Review of Educational Research. 1994; 64, 37–54.

[49] Lin SY, Cai J. Evaluation of environmental education in protected area based on visitors’ ecological literacy: a case study of Beijing Cuihu Wetland Park. Forestry and Ecological Sciences. 2019; 34 (4), 451–457. (In Chinese)

[50] McDaniel J, Alley KD. Connecting local environmental knowledge and land use practices: a human ecosystem approach to urbanization in West Georgia. Urban Ecosystems. 2005; 8 (1), 23–38.

[51] Hammarsten M, Askerlund P, Almers E, Avery H, Samuelsson T. Developing ecological literacy in a forest garden: children’s perspectives. Journal of Adventure Education and Outdoor Learning. 2018; 1–15. 10.1080/14729679.2018.1517371.

[52] Wells CN, Hatley M, Walsh J. Planting a native pollinator garden impacts the ecological literacy of undergraduate students. The American Biology Teacher. 2021; 83 (4), 210–213.

[53] Kragh G, Stafford R, Curtin S, Diaz A. Environmental volunteer well-being: managers’ perception and actual well-being of volunteers. F1000Research. 2016; 5, 2679. 10.12688/f1000research.10016.1. 28184285 PMC5288684

[54] Winch K, Stafford R, Gillingham P, Thorsen E, Diaz A. Diversifying environmental volunteers by engaging with online communities. People and Nature. 2021; 3, 17–31.

[55] Rudd JR, Pesce C, Strafford BW, Davids K. Physical Literacy—A Journey of Individual Enrichment: An Ecological Dynamics Rationale for Enhancing Performance and Physical Activity in All. Frontiers in Psychology. 2020; 11: 1904. doi: 10.3389/fpsyg.2020.01904 32849114 PMC7399225

