# Supplementary material for: The effectiveness of lifestyle interventions on ecological literacy: A contribution to the underlying mechanism in linguistic ecology
Source: PLoS One. 2023 Jun 29;18(6):e0287286. doi: 10.1371/journal.pone.0287286 (PMC10310022; doi:10.1371/journal.pone.0287286)
Supplement: S1 File — (ZIP) [file pone.0287286.s002.zip › S1 Questionnaire.docx]

**S1 Questionnaire (English)**

1. Do you agree that nature is very important in life?

A. Strongly agree B. Agree C. Not sure D. Disagree E. Strongly disagree

2. Do you agree that participating in outdoor activities is very important?

A. Strongly agree B. Agree C. Not sure D. Disagree E. Strongly disagree

3. “I am very interested in improving my ecological knowledge and understanding, as well as the level of ecological literacy”. Do you agree with this view?

A. Strongly agree B. Agree C. Not sure D. Disagree E. Strongly disagree

4. What is the frequency of your average daily outdoor activity every week?

A. ≧7 B. 5-6 C. 3-4 D. 1-2 E. Not at all

5. What is the frequency of your main activities in ecological areas such as forest parks, wetland parks, and nature reserves?

A. >2 (a week) B. 1-2 (a week) C. 1-2 (a month) D. 1-6 (a year) E. Not at all

1. In the past five years, how often have you participated in volunteer activities related to ecological environmental protection?

A. Always B. Often C. Sometimes D. Hardly E. Never

7. How often do you use ecological knowledge in your study or work?

A. Always B. Often C. Sometimes D. Hardly E. Never

**S1 Questionnaire (Chinese)**

1. 您认为自然在生活中是否重要 。
2. 非常重要 B. 比较重要 C. 一般

D. 不重要 E. 完全不重要

2. 您认为经常参加户外活动是否重要 。

A. 非常重要 B. 比较重要 C. 一般

D. 不重要 E. 完全不重要

3. 您对提高自己的生态知识与理解能力以及生态素养水平是否感兴趣 。

A. 非常感兴趣 B. 比较感兴趣 C. 不太确定

D. 不太感兴趣 E. 完全不感兴趣

4. 您平均每周日常户外活动的频率是 。

A. 7次及以上 B. 5-6次 C. 3-4次

D. 1-2次 E. 几乎不活动

1. 您主要在森林公园、湿地公园、自然保护区等生态区域活动的频率是 。
2. 每周2次以上 B. 每周1-2次 C. 每月1-2次

D. 每年几次（1-6次左右） E. 几乎不活动

6. 在过去五年内，您参与的保护生态环境相关志愿者活动的频率是 。

A. 总是参加 B. 经常参加 C. 有时参加

D. 偶尔参加 E. 完全不参加

7. 您在学习或工作中使用生态知识的频率是 。

A. 总是使用 B. 经常使用 C. 有时使用

D. 偶尔使用 E. 完全不使用
